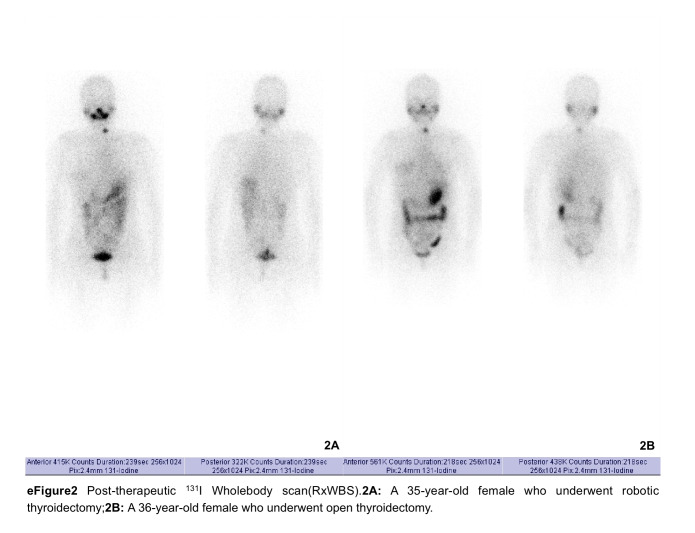# Observational cohort study on safety and efficacy of robotic thyroidectomy with super-meticulous capsular dissection versus open surgery for thyroid cancer: postoperative dynamic risk assessment of radioactive iodine therapy

**DOI:** 10.1097/JS9.0000000000002071

**Published:** 2024-09-12

**Authors:** Xiangquan Qin, Yufan Zhang, Jia Luo, Lingjuan Zeng, Xia Liu, Ting Zhang, Lin Ren, Linjun Fan, Dingde Huang

**Affiliations:** a Department of Breast and Thyroid Surgery, The Southwest Hospital of Army Military Medical University; b Department of Nuclear Medicine, The Southwest Hospital of Army Military Medical University; c Department of Anesthesiology, The Southwest Hospital of Army Military Medical University, Shapingba District, Chongqing, People’s Republic of China

**Keywords:** 131I-WBS, hypoparathyroidism, RAI, robot thyroidectomy, thyroid cancer

## Abstract

**Objective:**

To assess the efficacy and safety of robotic thyroidectomy (RT) with super-meticulous capsular dissection (SMCD) versus open thyroidectomy (OT), the authors used a dynamic risk assessment system incorporating ^131^I-WBS along with radioactive iodine (RAI) efficacy evaluation.

**Background:**

Currently, the therapeutic efficacy of robotic surgery remains controversial. The ^131^I whole-body scan (^131^I-WBS) dynamic risk assessment system can detect small residual thyroid tissues and lesions, which may be used as indicators for the surgical efficacy of RT or OT thyroidectomy in differentiated thyroid cancer (DTC).

**Methods:**

This retrospective cohort study included 2349 patients who underwent total thyroidectomy followed by RAI therapy in our department between August 2017 and June 2023. Propensity score matching was performed at a ratio of 1:3 based on surgical type and mean follow-up duration to minimize selection bias after excluding those lost to follow-up. The primary outcome was surgical completeness, assessed using a dynamic risk system incorporating ^131^I-WBS along with RAI efficacy evaluation.

**Results:**

There was no significant difference in the number of metastatic lymph nodes removed between the two groups (*P*=0.45). The incidence rate of parathyroid gland transplantation was 395 (68.7%) in the OT group and 8 (3.8%) in the RT group (*P*<0.001). There were no differences in the thyroidectomy completeness based on the 3 h iodine uptake rate and ^99m^TcO_4_^−^ thyroid imaging between the two groups. The dynamic risk assessment with and without ^131^I-WBS showed significant differences (*P*<0.001). The postoperative and post-RAI dynamic risk scores, evaluated at the time of RAI and 6 months after RAI, did not differ significantly between the two groups (*P*>0.05). The rates of transient and permanent hypoparathyroidism were higher in the OT group than in the RT group (*P*<0.05). The local recurrence rates showed no significant difference between the groups.

**Conclusions:**

This study demonstrates that RT with SMCD can achieve outcomes equivalent to those of traditional open surgery when integrating the ^131^I-WBS dynamic evaluation system and the therapeutic effects of RAI. Additionally, robot surgery demonstrated a notable advantage in protecting parathyroid function.

## Introduction

Highlights

- We introduced a novel approach by integrating ^131^I-WBS into a dynamic risk assessment framework, coupled with a postoperative evaluation of RAI therapy efficacy. This innovative method provided a nuanced understanding of the comparative efficacy and safety of RT and OT, offering essential insights for clinical decision-making.
- Addressing a critical gap in the literature, we elucidated whether robot-assisted total thyroidectomy utilizing the super-meticulous capsular dissection (SMCD) technique could achieve outcomes comparable to open surgery. Our study marks the first attempt to dispel doubts surrounding the efficacy of RT in this context.
- Importantly, our findings confirm that RT can achieve thorough removal of thyroid tissue and tumor lesions akin to OT, while demonstrating lower rates of hypoparathyroidism and higher surgical safety. This underscores the potential of robotic total thyroidectomy as a viable alternative to open surgery for thyroid cancer patients.

The incidence of thyroid cancer is steadily increasing, with differentiated thyroid carcinoma (DTC) being the most prevalent type, accounting for over 90% of the cases^1^. Currently, the standard treatment for DTC involves surgical resection, radioactive iodine (RAI) therapy, and thyroid hormone suppression therapy. Surgical intervention, including traditional open, endoscopic, and robot-assisted surgery^2^, remains the cornerstone of treatment.

Endoscopic surgery allows access to the surgical site through remote and less visible areas beyond the neck, maximizing the preservation of neck aesthetics^3^. Consequently, it has found widespread applications in clinical practice^4^. Robotic surgical systems represent a more advanced form of endoscopic surgery, offering several advantages, including a more than 10 times magnified three-dimensional field of view, Endo-Wrist instruments surpassing human dexterity, robotic arm assistance, and remote-control capabilities^5^, and these features enable finer and more convenient operations compared with conventional endoscopic surgery^6^, making robotic surgery an important technique in thyroid surgery^7^. In particular, the super-meticulous capsular dissection (SMCD) technique previously explored by our team for robotic thyroidectomy allows for better preservation of the parathyroid glands and their blood supply by preserving the true capsule on the dorsal side of the thyroid, thus significantly reducing the incidence of transient and permanent hypoparathyroidism after surgery^7–9^. While robotic thyroid surgery is generally considered superior to endoscopic surgery, there is an ongoing debate regarding its efficacy and safety compared with open surgery^10,11^.

Due to the slow progression and favorable prognosis of DTCs, short-term follow-ups often fail to reveal significant differences in survival or recurrence rates among various treatment modalities^12^. Therefore, the evaluation of surgical outcomes for DTC relies primarily on the metrics including the number of lymph nodes cleared, postoperative serum thyroglobulin (Tg) levels, and follow-up imaging modalities, such as ultrasound and computed tomography (CT) scans^8^. However, these indicators may overlook tiny residual or micro-metastatic lesions of thyroid cancer, leading to inaccurate disease assessment. ^131^I whole-body scans (^131^I-WBS) after DTC surgery can detect small lesions overlooked by other imaging modalities, such as CT^13^ or ultrasound^14^, allowing for more accurate tumor risk stratification and assessment, thus facilitating a more objective evaluation of surgical outcomes^15^. Moreover, the complete removal of thyroid tissue and tumors is crucial for the effectiveness of postoperative RAI treatment^16^, as the extent of surgical resection directly influences RAI treatment efficacy. Thus, theoretically, the effectiveness of postoperative RAI treatment can serve as an important indicator for surgical outcomes.

The present study integrated ^131^I-WBS into a dynamic risk assessment system to enhance the precision for evaluating the efficacy of robotic total thyroidectomy (RT). This was the first study to utilize dynamic risk assessment and the effectiveness of RAI treatment as evaluation metrics, aiming to provide a more accurate assessment of the disparities between the efficacy of RT and open thyroidectomy (OT) for DTC.

## Methods

This study has been reported in accordance with the STROCSS (Supplemental Digital Content 4, http://links.lww.com/JS9/D428) criteria^17^. Data were retrospectively collected from patients who underwent total thyroidectomy followed by RAI therapy at our department between August 2017 and June 2023 (eFigure 1, Supplemental Digital Content 1, http://links.lww.com/JS9/D425).

To minimize selection bias related to surgical proficiency, only cases from surgical teams with the highest volume of thyroid cancer surgeries were included (OT: >500 cases/year, RT: >300 cases/year). The inclusion criteria were: 1) age of 18–65 years; 2) pathologically-confirmed DTC; 3) maximum lesion diameter ≤4 cm; and 4) undergoing total thyroidectomy and postoperative RAI therapy. The exclusion criteria were: 1) undergoing secondary surgery; 2) history of neck radiation therapy; 3) tumors found to invade critical structures, such as the esophagus, recurrent laryngeal nerve, major neck vessels, or trachea, on preoperative or intraoperative examination; 4) undergoing lateral neck dissection; and 5) preoperative routine examination revealing distant metastases. Details of the surgical procedures, patient selection flowchart, and RAI treatment are provided as online-only supplementary material (eMethods) (Supplemental Digital Content 2, http://links.lww.com/JS9/D426).

### Evaluation of surgical completeness

The completeness of both surgical procedures was compared by evaluating the amount of residual thyroid tissue and dynamic risk assessment during follow-up. Remnant thyroid tissue was assessed using the 3 h iodine uptake rate and ^99m^TcO_4_^−^ thyroid imaging at the time of RAI. The patients were orally administered 185–370 kBq (5–10 μCi) of ^131^I-NaI solution. After 3 h, the radioactivity count of the thyroid region was measured using a thyroid function meter. The iodine uptake rate was calculated using the formula:[(thyroid region count − background count)/(standard source count − background count)]×100%.

For ^99m^TcO_4_^−^ thyroid imaging, ~15 min after intravenous administration of about 74–185 MBq (2–5 mCi) of ^99m^Tc-pertechnetate, anterior neck images were acquired using a gamma camera equipped with a high-resolution parallel-hole collimator (Symbia T6, Siemens). Imaging involved a 20% window centered around the 140 KeV peak of ^99m^Tc and a 128×128 computer matrix. Two experienced nuclear medicine physicians interpreted the images, with negative results indicating no uptake within the thyroid bed and positive results indicating any uptake above the background levels within the thyroid bed.

Dynamic risk assessment was conducted at the time of RAI and 6 months after RAI therapy, including all clinical, biochemical Tg and thyroglobulin antibodies (TgAb), and imaging (^131^I-WBS, CT, and ultrasound) findings obtained during follow-up. Post-therapeutic ^131^I whole-body scans (RxWBS) were acquired 48 h after RAI therapy. Diagnostic ^131^I whole-body scans (DxWBS) with 74 MBq ^131^I and neck ultrasonography were performed 6 months later. Tg, TgAb, and thyroid-stimulating hormone (TSH) levels were routinely measured through chemiluminescence during the follow-up.

According to the 2015 American Thyroid Association (ATA) guidelines^18^, dynamic risk assessment classifications included excellent response (ER; TSH-stimulated Tg <1 ng/ml or suppressed Tg <0.2 ng/ml in the absence of structural or functional evidence of disease), biochemical incomplete response (BIR; TSH-stimulated Tg ≥10 ng/ml or suppressed Tg ≥1 ng/ml in the absence of structural or functional evidence of disease, including patients with rising anti-Tg antibody levels), structural incomplete response (SIR; structural or functional evidence of disease with any Tg level, with or without anti-Tg antibodies), and indeterminate response (IDR; 1 ng/ml ≤ TSH-stimulated Tg <10 ng/ml or 0.2 ng/ml ≤ suppressed Tg <1 ng/ml in the absence of structural or functional evidence of disease, including patients with stable or declining anti-Tg antibody levels). Scores of 1, 2, 3, and 4 were assigned to ER, IDR, BIR, and SIR, respectively (eFigure 2A and 2B, Supplemental Digital Content 3, http://links.lww.com/JS9/D427, Tables 3 and 4).

### Complications

Any episodes of tetany, transient and permanent hypoparathyroidism^19^, temporary and permanent hoarseness^20^, and postoperative bleeding^21^ were documented (Table 5).

### Statistical analyses

The study’s sample size was determined based on eligibility criteria, including histological diagnosis and year of treatment. Propensity Score Matching (PSM) analysis was used to assess differences in baseline patient characteristics between the two groups. Continuous variables were presented as means with SD, while categorical variables were presented as frequencies and proportions. Statistical analyses were conducted using SPSS 27 (IBM Corp.). Procedure videos were generated using Final Cut Pro 2023 (Apple Inc.), and figures were created using Photoshop 2023 (Adobe Inc.).

## Results

### Baseline characteristics before and after PSM

Before PSM, 2082 patients underwent OT treatment, while 267 patients underwent RT treatment. Among them, 1342 (64.5%) cases in the OT group and 119 (44.6%) cases in the RT group underwent total thyroidectomy (TT) + unilateral central neck dissection (UCND). Additionally, 740 (35.5%) cases underwent TT + bilateral central neck dissection (BCND) in the OT group, compared with 148 (55.4%) cases in the RT group. Both comparisons showed statistically significant differences (*P*<0.001). The median follow-up times for the OT and RT groups were 34 months and 40 months, respectively, with a statistically significant difference (*P*<0.001).

PSM was performed for the two groups of patients treated with OT and RT in a 3:1 ratio based on the surgical type and mean follow-up duration. After matching, the proportions of TT + UCND were 217 (37.7%) and 82 (38.7%), while those for TT + BCND were 358 (62.3%) and 130 (61.3%) in the two groups, respectively, with no statistically significant differences (*P*=0.81). The median follow-up time was 31 months and 33 months, respectively, with no statistically significant difference (*P*=0.41) (Table 1).

**Table 1:** Baseline characteristics of patients before and after propensity score matching (PSM)

	Before PSM, No. (%)			After PSM, No. (%)		
	OT group (*N*=2082)	RT group (*N*=267)	*P*	OT group (*N*=575)	RT group (*N*=212)	*P*
Operation type			<0.001			0.81
TT + UCLN	1342 (64.5)	119 (44.6)		217 (37.7)	82 (38.7)	
TT + BCLN	740 (35.5)	148 (55.4)		358 (62.3)	130 (61.3)	
Mean follow-up (months)	34 (7–77)	40 (7–78)	<0.001	31 (7–77)	33 (7–75)	0.41

In the matched groups after PSM, the mean ages for the OT and RT groups were 42.89±10.54 years and 39.33±10.37 years, respectively, with a significant difference between the groups (*P*<0.001). The mean diameters of the largest tumor nodules detected by ultrasound were 12.92±7.10 mm and 12.97±7.58 mm for the OT and RT groups, respectively, with no statistically significant difference between the groups (*P*=0.94). Additionally, the mean operative times for TT + UCND were 112.59±34.77 min and 134.40±30.92 min, while those for TT + BCND were 118.48±34.19 min and 142.58±34.44 min for OT and RT, respectively, with significant differences between the groups (*P*<0.001). The number of dissected lymph nodes in the OT group was significantly higher than that in the RT group (12.99±9.00 vs. 10.02±5.96, *P*<0.001). However, there was no significant difference in the number of metastatic lymph nodes removed between the two groups (3.80±3.55 vs. 3.59±3.04, *P*=0.45). Furthermore, the incidence rates of parathyroid gland transplantation were 395 (68.7%) and 8 (3.8%) in the OT and RT groups, respectively, exhibiting a significant difference (*P*<0.001). There were no differences in sex, marital status, comorbidities, smoking, alcohol consumption, and intraoperative blood loss between the two groups (*P*>0.05) (Table 2).

**Table 2:** Demographic characteristics, surgical procedures, and perioperative characteristics

	OT group (*N*=575)	RT group (*N*=212)	*P*
Age, years	42.89±10.54	39.33±10.37	<0.001
Sex			0.40
Female	414 (72.0)	159 (75.0)	
Male	161 (28.0)	53 (25.0)	
Marital status			0.06
Unmarried	44 (7.7)	26 (12.3)	
Married	520 (90.4)	178 (83.9)	
Divorced	8 (1.4)	7 (3.3)	
Widow	3 (0.5)	1 (0.5)	
BMI (kg/m^2^)	24.31±3.34	23.657±3.52	0.02
Comorbidity			
Hypertension	68 (11.8)	25 (11.8)	0.99
Diabetes mellitus	22 (3.8)	9 (4.2)	0.79
Smoking			0.09
Yes	68 (11.8)	16 (7.5)	
No	507 (88.2)	196 (92.5)	
Alcohol			0.05
Yes	91 (15.8)	22 (10.4)	
No	484 (84.2)	190 (89.6)	
Sonographic tumor diameter (mm)	12.92±7.10	12.97±7.58	0.94
T (cm)			0.48
0–2	499 (86.8)	188 (88.7)	
2–4	76 (13.2)	24 (11.3)	
N			0.65
N0	69 (12.0)	28 (13.2)	
N1	506 (88.0)	184 (86.8)	
Tumor location			0.06
Unilateral	381 (66.3)	135 (63.7)	
Bilateral	180 (31.3)	77 (36.3)	
Isthmus	14 (2.4)	0	
Hashimoto’s thyroiditis			0.06
Yes	134 (23.3)	36 (17.0)	
No	441 (76.7)	176 (83.0)	
Operative time (min)			
TT + UCND	112.59±34.77	134.40±30.92	<0.001
TT + BCND	118.48±34.19	142.58±34.44	<0.001
Operation type			0.81
TT + UCND	217 (37.7)	82 (38.7)	
TT + BCND	358 (62.3)	130 (61.3)	
Parathyroid gland mis-resection			<0.001
Yes	395 (68.7)	8 (3.8)	
No	181 (31.5)	204 (96.2)	
Number of parathyroid glands transplanted			<0.001
0	180 (31.3)	204 (96.2)	
1	244 (42.4)	7 (3.3)	
2	135 (23.5)	1 (0.5)	
3	15 (2.6)	0	
4	1 (0.2)	0	
Estimated blood loss (ml)	30.05±17.02	29.84±20.93	0.88
Number of dissected lymph nodes	12.99±9.00	10.02±5.96	<0.001
Number of metastatic lymph nodes	3.80±3.55	3.59±3.04	0.45
Mean follow-up (months)	31 (7–77)	33 (7–75)	0.41

Data are the mean±SD, number (%) or median (min, max)

TT + BCND, Total thyroidectomy + bilateral central lymph nodes dissection; TT + UCND, Total thyroidectomy + unilateral central lymph node dissection.

### Evaluation of surgical efficacy

The differences in dynamic risk assessment with and without ^131^I-WBS are presented in Table 3. The inclusion of ^131^I-WBS altered the evaluation results in both OT and RT groups. A comparison of data before and after ^131^I-WBS incorporation into the dynamic risk assessment revealed a significant increase in IDR [OT group: 426 (74.09%) vs. 278 (48.35%), RT group: 150 (70.76%) vs. 100 (47.17%)] and SIR [OT group: 99 (17.22%) vs. 8 (1.39%), RT group: 37 (17.45%) vs. 3 (1.42%)] patients, along with a significant decrease in ER [OT group: 14 (2.43%) vs. 247 (42.96%), RT group: 6 (2.83%) vs. 84 (39.62%)] (Table 3).

**Table 3:** Postoperative dynamic risk assessment with/without ^131^I-WBS

	With ^131^I-WBS	Without ^131^I-WBS	*P*
OT (*N*=575)			<0.001
ER	14 (2.43)	247 (42.96)	
IDR	426 (74.09)	278 (48.35)	
BIR	36 (6.26)	42 (7.30)	
SIR	99 (17.22)	8 (1.39)	
RT (*N*=212)			<0.001
ER	6 (2.83)	84 (39.62)	
IDR	150 (70.76)	100 (47.17)	
BIR	19 (8.96)	25 (11.79)	
SIR	37 (17.45)	3 (1.42)	

BIR, biochemical incomplete response; ER, excellent response; IDR, indeterminate response; SIR, structural incomplete response.

A comparison of completeness of surgical procedures between the OT and RT groups showed no differences in thyroidectomy completeness based on the 3 h iodine uptake rate (1.48±0.60 vs. 1.61±0.87, *P*>0.05) and ^99m^TcO_4_^−^ thyroid imaging [459 (79.83%) vs. 157 (74.06%), *P*>0.05]. Additionally, the dynamic risk assessment evaluated at the time of RAI and 6 months after RAI did not significantly differ between the two groups. Furthermore, the postoperative and post-RAI dynamic risk scores were similar between the two groups, indicating similar responses to surgery and RAI treatment (Table 4).

**Table 4:** Surgical completeness parameters of 787 patients at 3.7–5.55 GBq RAI ablation therapy

	OT group (*N*=575)	RT group (*N*=212)	*P*
3 h Iodine uptake rate (%)	1.48±0.60	1.61±0.87	0.21
^99m^TcO_4_- thyroid imaging			0.08
Positive	116 (20.17)	55 (25.94)	
Negative	459 (79.83)	157 (74.06)	
Postoperative dynamic risk assessment			0.55
ER	14 (2.43)	6 (2.83)	
IDR	426 (74.09)	150 (70.76)	
BIR	36 (6.26)	19 (8.96)	
SIR	99 (17.22)	37 (17.45)	
Post-RAI dynamic risk assessment			0.70
ER	396 (68.87)	144 (67.92)	
IDR	105 (18.26)	45 (21.23)	
BIR	31 (5.39)	11 (5.19)	
SIR	17 (2.96)	5 (2.36)	
NA	26 (4.52）	7 (3.30)	
Postoperative dynamic risk score	2.38±0.79	2.41±0.81	0.67
Post-RAI dynamic risk score	1.40±0.70	1.40±0.73	0.96

Data are means±SD/SEM or number (%).

BIR, biochemical incomplete response (score=3); ER, excellent response (score=1); IDR, indeterminate response (score=2); NA, not applicable; RAI, radioactive iodine; SIR, structural incomplete response (score=4).

### Complications and local recurrence rates

The occurrence rates of tetany in the OT and RT groups were 214 cases (37.2%) and 1 case (0.5%), while the incidence rates of transient hypoparathyroidism were 393 cases (68.3%) and 67 cases (31.6%), respectively, indicating significant differences between the two groups (*P*<0.001). Similarly, the rates of permanent hypoparathyroidism were 18 cases (3.1%) and 1 case (0.5%), respectively, representing a statistically significant difference (*P*=0.03). There were no differences between the two groups in the occurrence rates of postoperative hematoma, bleeding, and transient or permanent hoarseness. During the follow-up period, the local recurrence rates were 27 cases (4.7%) and 7 cases (3.3%) in the OT and RT groups, respectively, with no significant differences between the groups (*P*>0.05). Moreover, neither group exhibited distant metastases (Table 5).

**Table 5:** Postoperative complications and recurrence

	OT group (*N*=575)	RT group (*N*=212)	*P*
Hematoma (observed)	6 (1.0)	1 (0.5)	0.45
Tetany	214 (37.2)	1 (0.5)	<0.001
Transient hypoparathyroidism^a^	393 (68.3)	67 (31.6)	<0.001
Permanent hypoparathyroidism^a^	18 (3.1)	1 (0.5)	0.03
Temporary hoarseness^b^	12 (2.1)	1 (0.5)	0.12
Permanent hoarseness^b^	5 (0.9)	0	0.66
Lymphorrhagia	9 (1.6)	0	0.07
Postoperative infection	4 (0.7)	2 (0.9)	0.72
Transient hypocalcemia	327 (56.9)	126 (59.4)	0.52
Postoperative bleeding^c^	11 (1.9)	1 (0.5)	0.14
Extubation	1 (0.2)	0	0.54
Recurrence	27 (4.7)	7 (3.3)	0.39

Data are presented as numbers (%).

^a^ ‘Transient hypoparathyroidism’ was defined as a serum PTH level below the normal range on the first postoperative day. However, if the PTH level failed to recover within 6 months after surgery, it was considered permanent hypoparathyroidism.

^b^ ‘Temporary hoarseness’ referred to a voice change 6 months after surgery due to recurrent laryngeal nerve injury. If hoarseness did not fully recover within 6 months after surgery and was confirmed by laryngoscopy as vocal-cord paralysis, it was defined as ‘permanent recurrent laryngeal nerve injury’.

^c^ ‘Postoperative bleeding’ referred to bleeding at the surgical site or subcutaneous tunnel area requiring further surgical intervention to control the bleeding or clear any subcutaneous hematomas.

## Discussion

Currently, open surgery remains the established standard treatment for thyroid cancer. However, it often results in noticeable neck appearance changes postoperatively, which can be particularly distressing, especially for young female patients, potentially leading to lifelong regret^22^. The da Vinci robotic system, as an advanced endoscopic control device, has emerged as an alternative in thyroid surgery^23^. Nevertheless, there is an ongoing debate regarding whether the therapeutic outcomes of robotic surgery are comparable to those of open surgery^11^. In this study, we introduced dynamic risk assessment with ^131^I-WBS and assessed the efficacy of RAI therapy to compare surgical outcomes between the RT with SMCD and OT groups. Our findings revealed no significant differences in the results of dynamic risk assessment before and after RAI therapy between the two groups, further confirming that the completeness of lesion resection in the RT group was similar to that of open surgery.

Analysis of demographic data revealed that patients in the RT group were, on average, younger compared with those in the OT group. This suggests that the scarless postoperative neck appearance associated with the RT approach provides better cosmetic outcomes and may be preferred by younger patients. Tumor diameters, staging, and locations were similar between the two groups, indicating similar tumor burdens and surgical complexities. However, the RT group had longer operative times for both TT + UCND and TT + BCND compared with the OT group. We attribute this primarily to the preparation time required for robotic surgery, including trocar placement, establishment of the initial operative space, and robotic arm connection, which typically takes about 30 min^24^. This resulted in longer overall operative times compared with open surgery, in line with previous research findings^6^.

Surgical efficacy evaluations often rely on single-point static assessments based on intraoperative or postoperative pathological and clinical data^25^. However, these assessments lack the ability to dynamically adjust based on clinical data obtained during follow-up, limiting their effectiveness. The ATA dynamic risk stratification system, incorporating biochemical (Tg and TgAb) and imaging (^131^I-WBS, CT, and ultrasound) findings, provides a more comprehensive framework for assessment^26^. ^131^I-WBS, a routine imaging modality after RAI therapy, can be used to detect tiny residual or micro-metastatic thyroid cancer lesions often missed by other structural imaging modalities, including CT and ultrasound. Our study revealed a significant difference in dynamic risk assessment with and without ^131^I-WBS. Positive findings on ^131^I-WBS led many patients initially classified as ER to be reclassified as IDR or SIR. This highlights the importance of dynamic risk assessment with ^131^I-WBS for accurate evaluation of surgical efficacy.

Furthermore, we assessed the surgical completeness of both methods by comparing multiple parameters. Although more lymph nodes were cleared in the OT group than in the RT group, there was no significant difference in the number of metastatic lymph nodes between the two groups. Additionally, high iodine uptake and increased radioactivity in the thyroid bed during technetium imaging and ^131^I-WBS provided evidence of remnant thyroid tissue. Through 3 h iodine uptake, technetium imaging, and dynamic risk assessment with ^131^I-WBS at the time of RAI and after 6 months of RAI therapy, the RT group demonstrated outcomes similar to the OT group. This indicated that RT was as effective as OT in tumor eradication.

Besides, the rates of parathyroid gland mis-resection and transplantation were lower in the RT group compared with the OT group. This may be attributed to the magnified 3D field of view and flexibility of Endo-Wrist instruments in robotic surgery, which facilitated finer operations. Moreover, the RT group exhibited significantly lower rates of transient and permanent hypoparathyroidism compared with the OT group, which was consistent with previous reports from our team^7^. We consider that this was due to better protection of the parathyroid gland by preserving the true capsule behind the thyroid gland through SMCD. Previous studies have demonstrated that the SMCD technique^7^ in robotic surgery effectively preserves challenging-to-retain parathyroid gland types, including compact, and subcapsular parathyroid glands, along with their blood supply^27^, thereby reducing the incidence of parathyroid gland dysfunction^9^. Finally, during follow-up, ranging from 6 to 76 months, no difference was found in the recurrence rate between the two groups.

### Strengths and limitations

The present study had several strengths. Firstly, we uniquely integrated ^131^I-WBS into a dynamic risk assessment framework, complemented by a postoperative evaluation of RAI therapy efficacy. This innovative method offered a nuanced understanding of the comparative efficacy and safety of RT and OT, providing crucial insights for treatment decision-making. Secondly, PSM allowed meticulous mitigation of biases arising from clinical and pathological variations between the two groups, enhancing the reliability and credibility of our conclusions and ensuring robust comparisons between the two approaches. Thirdly, the inclusion of a large and diverse observational cohort, reflecting real-world clinical practice, improved the generalizability of our findings. Through comprehensive long-term follow-up assessments, we not only recorded immediate postoperative outcomes but also elucidated the enduring impact of RT and OT on factors such as recurrence rates and parathyroid function. This holistic perspective offers valuable insights into the sustained efficacy and patient-centered outcomes associated with each surgical modality. Finally, our study addressed a critical gap in current research by comparing the safety and efficacy of RT and OT specifically for thyroid cancer, contributing significantly to informed clinical decision-making and ultimately improving patient outcomes and quality of life.

However, the study also had several limitations. Firstly, the proficiency of RT in a single center may not represent the overall level for thyroid surgery. Moreover, the study used the unilateral axillo-breast approach or bilateral axillo-breast approach for RT, which may have different effects compared with other approaches. Secondly, patients requiring lateral neck dissections were not included, necessitating further research into the effectiveness of robot-assisted surgery for this subgroup. Thirdly, DTC generally has a favorable prognosis, and the limited follow-up time in this study may not reflect final treatment outcomes for all cases. Therefore, designing multicenter prospective studies with larger sample sizes, broader indications, and longer follow-up durations is essential to further validate the conclusions of this study.

## Conclusions

This study introduced a novel method by incorporating the dynamic assessment system of ^131^I-WBS with the efficacy of radioactive iodine therapy, allowing precise evaluation of the completeness of thyroid cancer surgery. Moreover, it indicated that RT through SMCD achieved efficacy comparable to OT while improving the protection of parathyroid function.

## Ethical approval

This study was approved by the Clinical Research Ethics Committee of Southwest Hospital, the Southwest Hospital of Army Military Medical University, Chongqing, China (approval number: KY2022214).

## Consent

Because this study is a retrospective cohort study, only patient data needs to be collected retrospectively, and through ethical review, we exempted patients from signing informed consent.

## Source of funding

We conducted this research without external funding.

## Author contribution

D.D.H. and L.J.F.: had full access to all the data in the study and took responsibility for the integrity of the data and the accuracy of the data analysis; Q.X.Q. and Y.F.Z.: contributed equally to this work; D.D.H., L.J.F., and Q.X.Q.: concept and design; Q.X.Q., Y.F.Z., J.L., X.L., T.Z., L.R., and J.J.Z.: collection data; Q.X.Q. and Y.F.Z.: acquisition, analysis, or interpretation of data; Q.X.Q. and Y.F.Z.: drafting of the manuscript; D.D.H. and L.J.F.: critical revision of the manuscript for important intellectual content; Q.X.Q. and Y.F.Z.: statistical analysis; D.D.H., L.J.Z., and L.J.F.: administrative, technical, or material support; D.D.H. and L.J.F.: supervision.

## Conflicts of interest disclosure

The authors declare that they have no financial conflicts of interest with regard to the content of this report.

## Research registration unique identifying number (UIN)

The present study is registered at www.clinicaltrials.gov (ChiCTR2400088020).

## Guarantor

Linjun Fan and Dingde Huang.

## Data availability statement

Datasets generated during and/or analyzed during the current study are publicly available.

## Provenance and peer review

Not commissioned, externally peer-reviewed.

## Supplementary Material

**Figure:**