# Supplementary figures and images for: Observational cohort study on safety and efficacy of robotic thyroidectomy with super-meticulous capsular dissection versus open surgery for thyroid cancer: postoperative dynamic risk assessment of radioactive iodine therapy
Source: Int J Surg. 2024 Sep 12;111(1):153–9. doi: 10.1097/JS9.0000000000002071 (PMC11745651; doi:10.1097/JS9.0000000000002071)

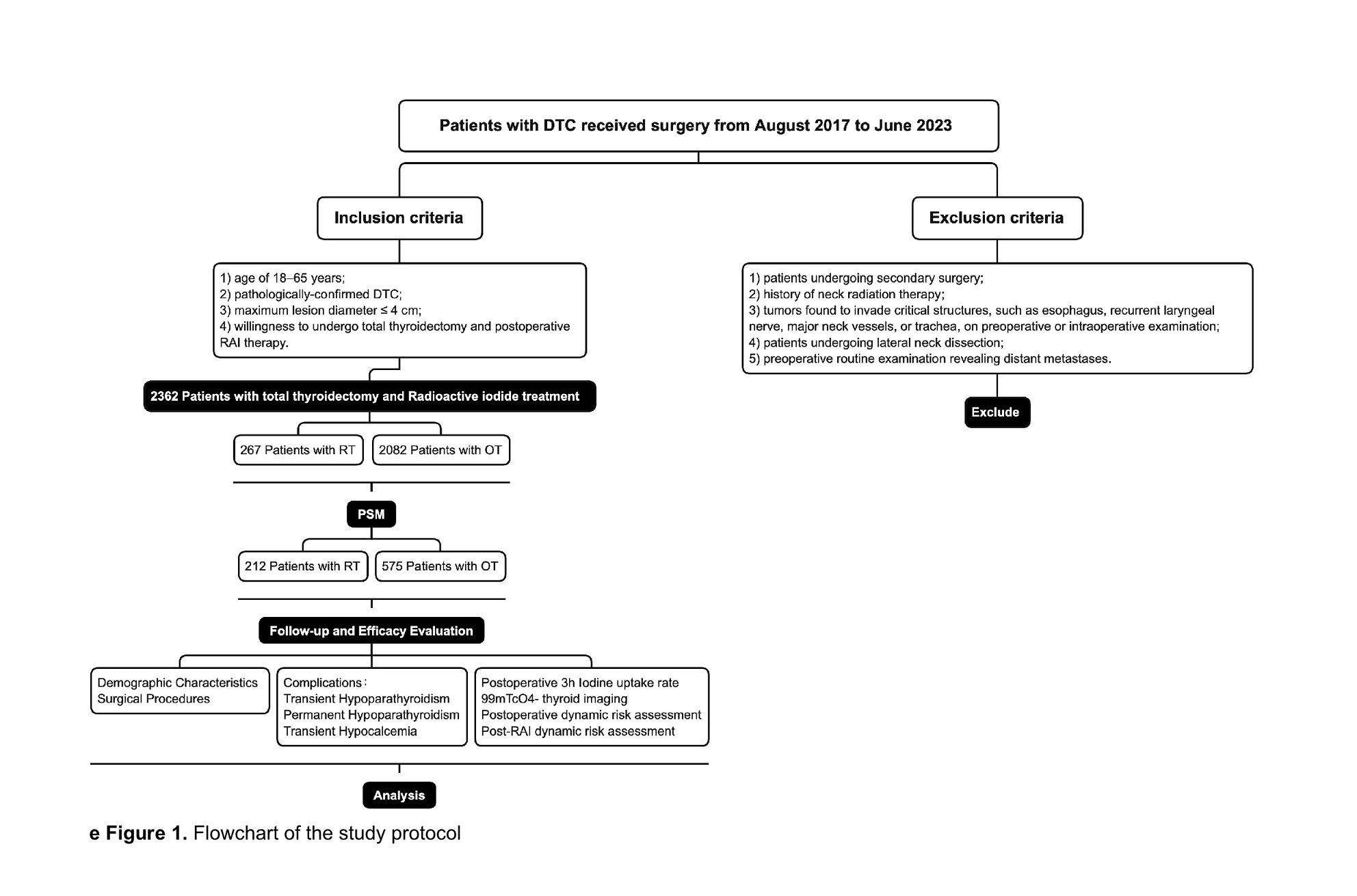

Supplement: Supplementary file 1 [file js9-111-0153-s001.jpg]
