# Supplementary material for: Observational cohort study on safety and efficacy of robotic thyroidectomy with super-meticulous capsular dissection versus open surgery for thyroid cancer: postoperative dynamic risk assessment of radioactive iodine therapy
Source: Int J Surg. 2024 Sep 12;111(1):153–9. doi: 10.1097/JS9.0000000000002071 (PMC11745651; doi:10.1097/JS9.0000000000002071)
Supplement: Supplementary file 2 [file js9-111-0153-s002.docx]

**eMethods**

**Surgical procedures** Both surgical procedures involved endotracheal intubation, general anesthesia administration, neck hyperextension, and routine disinfection and draping of the surgical site. We have previously outlined the OT procedure in detail^32^. OT involved a meticulous conventional dissection technique, closely adhering to the true thyroid capsule for resection^33^, ensuring parathyroid gland preservation, and exposing the recurrent laryngeal nerve. The RT procedure, including surgical access, free flaps, thyroidectomy, and central compartment dissection using the unilateral axillo-breast approach, has been extensively described in previous literature^8^. We used the super-meticulous capsular dissection technique, which is briefly outlined here. After severing the upper and lower blood vessels of the thyroid gland using an ultrasonic knife, the thyroid gland was dissected posteriorly using an electrocautery hook to preserve as much of the true capsule as possible. This allowed better protection of the parathyroid glands and their blood supply. If the parathyroid glands cannot be preserved in situ, they are immediately made into a saline suspension and injected into the muscle. In our study, the parathyroid tissue suspension was injected directly into the ipsilateral sternocleidomastoid muscle in OT group, and it was performed using the same method following percutaneous puncture under a robotic camera in the RT group.

**RAI treatment**

All patients in our study underwent RAI treatment ranging from 3.7 to 5.55 GBq (100–150 mCi)^34^ within 6 months after surgery. The patients were required to adhere to a low-iodine diet (<50 µg/d) for 4 weeks and discontinue levothyroxine 3 weeks before RAI treatment to stimulate endogenous thyroid-stimulating hormone (TSH) production. A serum TSH level of ≥30 mU/L is necessary for optimal therapeutic benefits. Before RAI therapy, the patients were explained its purpose, potential side effects, implementation procedure, and radiation safety precautions, and written informed consent was obtained.

**Figure Legends**

**eFigure 1.** Flowchart of the study protocol

**eFigure 2** Post-therapeutic ^131^I whole-body scan (RxWBS). **2A:** A 35-year-old female who underwent robotic thyroidectomy; **2B:** A 36-year-old female who underwent open thyroidectomy.
