# Supplementary material for: Observational cohort study on safety and efficacy of robotic thyroidectomy with super-meticulous capsular dissection versus open surgery for thyroid cancer: postoperative dynamic risk assessment of radioactive iodine therapy
Source: Int J Surg. 2024 Sep 12;111(1):153–9. doi: 10.1097/JS9.0000000000002071 (PMC11745651; doi:10.1097/JS9.0000000000002071)
Supplement: Supplementary file 3 [file js9-111-0153-s004.pdf]

The STROCSS 2021 Guideline		
**Item no.**	**Item description**	**Page**
**TITLE**		
1	**Title**<br><br>**Observational Cohort Study on Safety and Efficacy of Robotic Thyroidectomy vs. Open Surgery for Thyroid Cancer: Postoperative Evaluation Using Dynamic Risk Assessment of RAI Therapy**	1
**ABSTRACT**		
2a	**Introduction** – briefly describe:<br>• To assess the efficacy and safety of RT vs. OT, we used a dynamic risk assessment system incorporating ^{131}I-WBS along with radioactive iodine (RAI) efficacy evaluation.<br>• Currently, the therapeutic efficacy of robotic surgery remains controversial. The 131I whole-body scan (131I-WBS) dynamic risk assessment system can detect small residual thyroid tissues and lesions, which may be used as indicators for surgical efficacy of robotic (RT) or open (OT) thyroidectomy in differentiated thyroid cancer (DTC).	1
2b	**Methods** - briefly describe:<br>• This retrospective cohort study included 2,349 patients who underwent total thyroidectomy followed by RAI therapy in our department between August 2017 and June 2023. Propensity score matching was performed at a ratio of 1:3 based on surgical type and mean follow-up duration to minimize selection bias after excluding those lost to follow-up.<br>• The primary outcome was surgical completeness, assessed using a dynamic risk system incorporating 131I-WBS along with RAI efficacy evaluation.	1
2c	**Results** - briefly describe:<br>• Significant differences were observed in the mean operative time for total thyroidectomy (TT) + unilateral central neck dissection (UCND) and TT + bilateral central neck dissection (BCND) between the two groups ($P < 0.001$). There were no differences in the thyroidectomy completeness based on the 3-hour iodine uptake rate and 99mTcO4− thyroid imaging between the two groups. The dynamic risk assessment with and without 131I-WBS showed significant differences ($P < 0.001$). The postoperative and post-RAI dynamic risk scores, evaluated at the time of RAI and 6 months after RAI, did not differ significantly between the two groups ($P > 0.05$). The rates of transient and permanent hypoparathyroidism showed significant differences between the groups ($P < 0.05$).	2
2d	**Conclusion** - briefly describe:<br>• **This study demonstrated that RT could achieve outcomes equivalent to those of traditional open surgery when integrating the 131I-WBS dynamic evaluation system and the therapeutic effects of RAI. Additionally, robot surgery demonstrated a notable advantage in protecting parathyroid function.**	2
**INTRODUCTION**		
3	**Introduction** – comprehensively describe:<br>• The incidence of thyroid cancer is steadily increasing, with differentiated thyroid carcinoma (DTC) being the most prevalent type, accounting for over	2

	90% of the cases1. Currently, the standard treatment for DTC involves surgical resection, radioactive iodine (RAI) therapy, and thyroid hormone suppression therapy. Surgical intervention, including traditional open surgery, endoscopic surgery, and robot-assisted surgery2, remains the cornerstone of treatment.<br>• Endoscopic surgery allows access to the surgical site through remote and less visible areas beyond the neck, maximizing the preservation of neck aesthetics3. Consequently, it has found widespread applications in clinical practice4. Robotic surgical systems represent a more advanced form of endoscopic surgery, offering several advantages, including a more than 10 times magnified three-dimensional field of view, Endo-Wrist® instruments surpassing human dexterity, robotic arm assistance, and remote-control capabilities5. These features enable finer and more convenient operations compared to conventional endoscopic surgery6, making robotic surgery an important technique in thyroid surgery7. While robotic thyroid surgery is generally considered superior to endoscopic surgery, there is an ongoing debate regarding its efficacy and safety compared to open surgery8,9.<br>• Due to the slow progression and favorable prognosis of DTCs, short-term follow-ups often fail to reveal significant differences in survival or recurrence rates among various treatment modalities10. Therefore, the evaluation of surgical outcomes for DTC relies primarily on the metrics including the number of lymph nodes cleared, postoperative serum thyroglobulin (Tg) levels, and follow-up imaging modalities, such as ultrasound and computed tomography (CT) scans9. However, these indicators may overlook tiny residual or micro-metastatic lesions of thyroid cancer, leading to inaccurate disease assessment. 131I whole-body scans (131I-WBS) after DTC surgery can detect small lesions overlooked by other imaging modalities, such as CT11 or ultrasound12, allowing for more accurate tumor risk stratification and assessment, thus facilitating a more objective evaluation of surgical outcomes13. Moreover, the complete removal of thyroid tissue and tumors is crucial for the effectiveness of postoperative RAI treatment14, as the extent of surgical resection directly influences RAI treatment efficacy. Thus, theoretically, the effectiveness of postoperative RAI treatment can serve as an important indicator for surgical outcomes.<br>• The present study integrated 131I-WBS into a dynamic risk assessment system to enhance the precision for evaluating the efficacy of robotic total thyroidectomy (RT). This was the first study to utilize dynamic risk assessment and the effectiveness of RAI treatment as evaluation metrics, aiming to provide a more accurate assessment of the disparities between the efficacy of RT and open thyroidectomy (OT) for DTC.	
**METHODS**		
4a	**Registration**<br>• We conducted this research without external funding. This study was approved by the Clinical Research Ethics Committee of Southwest Hospital, the Southwest Hospital of Army Military Medical University, Chongqing, China (approval number: KY2022214).	Title page 1
4b	**Ethical approval**<br>• We conducted this research without external funding. This study was approved by the Clinical Research Ethics Committee of Southwest Hospital, the Southwest Hospital of Army Military Medical University, Chongqing, China (approval number: KY2022214).	Title page 1
4c	**Protocol**	3

	• This retrospective cohort study included 2,349 patients who underwent total thyroidectomy followed by RAI therapy in our department between August 2017 and June 2023. Propensity score matching was performed at a ratio of 1:3 based on surgical type and mean follow-up duration to minimize selection bias after excluding those lost to follow-up.<br>• The primary outcome was surgical completeness, assessed using a dynamic risk system incorporating 131I-WBS along with RAI efficacy evaluation.	
4d	**Patient and public involvement in research**<br>• Data were retrospectively collected from patients who underwent total thyroidectomy followed by RAI therapy at our department between August 2017 and June 2023(e Figure 1).<br>• To minimize selection bias related to surgical proficiency, only cases from surgical teams with the highest volume of thyroid cancer surgeries were included (OT: > 500 cases/year, RT: > 300 cases/year). The inclusion criteria were: 1) age of 18–65 years; 2) pathologically-confirmed DTC; 3) maximum lesion diameter ≤ 4 cm; and 4) undergoing total thyroidectomy and postoperative RAI therapy. The exclusion criteria were: 1) undergoing secondary surgery; 2) history of neck radiation therapy; 3) tumors found to invade critical structures, such as the esophagus, recurrent laryngeal nerve, major neck vessels, or trachea, on preoperative or intraoperative examination; 4) undergoing lateral neck dissection; and 5) preoperative routine examination revealing distant metastases. Details of the surgical procedures, patient selection flowchart, and RAI treatment are provided as online-only material supplementary (e Methods).	5
5a	**Study design**<br>• cohort,retrospective, single-centred	4
5b	**Setting and timeframe of research** – comprehensively describe:<br>• No. 30, Gaotan Yanzheng Street, Shapingba District, Chongqing, China.<br>• Department of Breast and Thyroid Surgery, Southwest Hospital, The First Affiliated Hospital of the Army Military Medical University,<br>• From March 2020 to March 2022,	5
5c	**Study groups**<br>• This retrospective cohort study included 2,349 patients who underwent total thyroidectomy followed by RAI therapy in our department between August 2017 and June 2023. Propensity score matching was performed at a ratio of 1:3 based on surgical type and mean follow-up duration to minimize selection bias after excluding those lost to follow-up.<br>• The primary outcome was surgical completeness, assessed using a dynamic risk system incorporating 131I-WBS along with RAI efficacy evaluation.	5
5d	**Subgroup analysis** – comprehensively describe:<br>• Continuous variables were summarized as means with standard deviation, while categorical variables were presented as frequencies and proportions. SF-36, NIS, VIS, SIS, and SCAR-Q data were transformed into total scale scores. Univariate and multivariate Cox proportional hazard regression models were used to evaluate the influence of variables on transient hypoparathyroidism. P < 0.05 (two-tailed) was considered statistically significant. Statistical analyses were performed using SPSS 27 (IBM Corp., Armonk, NY, USA). Videos for operative procedures were generated using Final Cut Pro 2023 (Apple, Cupertino,	5

	CA, USA), while figures were generated using Photoshop 2023 (Adobe, San Jose, CA, USA).	
6a	**Participants** – comprehensively describe:<br>• To minimize selection bias related to surgical proficiency, only cases from surgical teams with the highest volume of thyroid cancer surgeries were included (OT: > 500 cases/year, RT: > 300 cases/year). The inclusion criteria were: 1) age of 18–65 years; 2) pathologically-confirmed DTC; 3) maximum lesion diameter ≤ 4 cm; and 4) undergoing total thyroidectomy and postoperative RAI therapy. The exclusion criteria were: 1) undergoing secondary surgery; 2) history of neck radiation therapy; 3) tumors found to invade critical structures, such as the esophagus, recurrent laryngeal nerve, major neck vessels, or trachea, on preoperative or intraoperative examination; 4) undergoing lateral neck dissection; and 5) preoperative routine examination revealing distant metastases. Details of the surgical procedures, patient selection flowchart, and RAI treatment are provided as online-only material supplementary (e Methods)	4
6b	**Recruitment** – comprehensively describe:<br>• To minimize selection bias related to surgical proficiency, only cases from surgical teams with the highest volume of thyroid cancer surgeries were included (OT: > 500 cases/year, RT: > 300 cases/year). The inclusion criteria were: 1) age of 18–65 years; 2) pathologically-confirmed DTC; 3) maximum lesion diameter ≤ 4 cm; and 4) undergoing total thyroidectomy and postoperative RAI therapy. The exclusion criteria were: 1) undergoing secondary surgery; 2) history of neck radiation therapy; 3) tumors found to invade critical structures, such as the esophagus, recurrent laryngeal nerve, major neck vessels, or trachea, on preoperative or intraoperative examination; 4) undergoing lateral neck dissection; and 5) preoperative routine examination revealing distant metastases. Details of the surgical procedures, patient selection flowchart, and RAI treatment are provided as online-only material supplementary (e Methods)<br>• The completeness of both surgical procedures was compared by evaluating the amount of residual thyroid tissue and dynamic risk assessment during follow-up. Remnant thyroid tissue was assessed using the 3-hour iodine uptake rate and 99mTcO4− thyroid imaging at the time of RAI. The patients were orally administered 185–370 kBq (5–10 μCi) of 131I-NaI solution. After 3 hours, the radioactivity count of the thyroid region was measured using a thyroid function meter. The iodine uptake rate was calculated using the formula: [(thyroid region count − background count)/(standard source count − background count)] ×100%.<br>• For 99mTcO4− thyroid imaging, approximately 15 minutes after intravenous administration of about 74–185 MBq (2–5 mCi) of 99mTc-pertechnetate, anterior neck images were acquired using a gamma camera equipped with a high-resolution parallel-hole collimator (Symbia T6, Siemens, Germany). Imaging involved a 20% window centered around the 140 KeV peak of 99mTc and a 128 × 128 computer matrix. The images were interpreted by two experienced nuclear medicine physicians, with negative results indicating no uptake within the thyroid bed and positive results indicating any uptake above the background levels within the thyroid bed.<br>• Dynamic risk assessment was conducted at the time of RAI and 6 months after RAI therapy, including all clinical, biochemical Tg and Thyroglobulin antibodies(TgAb), and imaging (131I-WBS, CT, and ultrasound) findings	3-5

	obtained during follow-up. Post-therapeutic 131I whole-body scans (RxWBS) were acquired 48 hours after RAI therapy. Diagnostic 131I whole-body scans (DxWBS) with 74 MBq 131I and neck ultrasonography were performed 6 months later. Tg, TgAb, and TSH levels were routinely measured through chemiluminescence during the follow-up.<br>• According to the 2015 American Thyroid Association (ATA) guidelines16, dynamic risk assessment classifications included excellent response (ER; TSH-stimulated Tg <1 ng/mL or suppressed Tg <0.2 ng/mL in the absence of structural or functional evidence of disease), biochemical incomplete response (BIR; TSH-stimulated Tg ⩾10 ng/mL or suppressed Tg ⩾1 ng/mL in the absence of structural or functional evidence of disease, including patients with rising anti-Tg antibody levels), structural incomplete response (SIR; structural or functional evidence of disease with any Tg level, with or without anti-Tg antibodies), and indeterminate response (IDR; 1 ng/mL ⩽ TSH-stimulated Tg < 10 ng/mL or 0.2 ng/mL ⩽ suppressed Tg < 1 ng/mL in the absence of structural or functional evidence of disease, including patients with stable or declining anti-Tg antibody levels). Scores of 1, 2, 3, and 4 were assigned to ER, IDR, BIR, and SIR, respectively (e Figure 2A and 2B, tables 3 and 4).	
6c	**Sample size** – comprehensively describe:<br>• To minimize bias resulting from inadequate surgical experience of the operating physician, only surgeons who were members of a senior surgical team, with an annual volume of over 500 thyroid surgery cases, were included. Inclusion criteria for the RT group were: 1. Primary hyperthyroidism unresponsive to medical treatment; 2. Thyroid enlargement above the third degree; 3. High functional adenoma; 4. Benign thyroid tumors measuring > 4 cm or those causing neck deformity, compression symptoms, or significant short-term growth despite measuring < 4 cm. The exclusion criteria were: 1. Posterior sternal goiter; 2. Postoperative pathological diagnosis of malignant thyroid tumor; 3. Secondary surgical patients; 4. Other concomitant diseases not suitable for surgery3.<br>• Additionally, all patients completed evaluations using the Short Form (SF)-36 scale12, Voice Impairment Score (VIS)13, Swallowing Impairment Score (SIS), Neck Impairment Score (NIS)14, and Scar questionnaire (SCAR-Q) scale15,16. Any episodes of transient hypoparathyroidism, permanent hypoparathyroidism17, temporary hoarseness18, and postoperative bleeding19 were documented (Table 3). SF-36, a widely used health survey tool for evaluating HR-QoL, was administered to all patients12. Additionally, SCAR-Q15 was used to assess appearance, symptoms, and psychosocial parameters16 (Table 5). Impairments were assessed using the NIS, SIS, and VIS20 (Table 5).<br>The surgical procedures(e Figure 2A、2B、2C, e video1-2),<br>Postoperative comparison of two groups(e Figure 3A、3B) and relevant videos are provided as online-only material supplementary (e Methods).	3
**METHODS - INTERVENTION AND CONSIDERATIONS**			
7a	**Pre-intervention considerations** – comprehensively describe:<br>• Preoperative patient optimisation (e.g. weight loss, smoking cessation, glycaemic control etc.)<br>• Pre-intervention treatment (e.g. medication review, bowel preparation, correcting hypothermia/-volemia/-tension, mitigating bleeding risk, ICU care etc.)	3
7b	**Intervention** – comprehensively describe:	

	• none	
7c	**Intra-intervention considerations** – comprehensively describe:<br>• none	
7d	**Operator details** – comprehensively describe:<br>• none	
7e	**Quality control** – comprehensively describe:<br>• none	
7f	**Post-intervention considerations** – comprehensively describe:<br>• none	
8	**Outcomes** – comprehensively describe:<br>• Primary outcomes, including transient hypoparathyroidism, permanent hypoparathyroidism, and transient hypocalcemia<br>• Patients were evaluated by scales [Short Form (SF)-36, Visual Impairment Scale (VIS), Swallowing Impairment Scale (SIS), Neck Impairment Scale (NIS), Scar questionnaire (SCAR-Q)].Definition of outcomes<br>• Follow-up period for outcome assessment, divided by group	4
9	**Statistics** – comprehensively describe:<br>• Continuous variables are summarized as the mean and standard deviation. Categorical variables are presented as frequencies and proportions. Data from HR-QoL and NIS, VIS, SIS, and SCAR-Q were transformed into total scale scores. Univariate and multivariate Cox proportional hazard regression models were implemented to evaluate the influence of variables on permanent hypoparathyroidism. $p < 0.05$ (two-tailed) was considered significant. Statistical analyses were carried out using SPSS 27 (IBM, Armonk, NY, USA). Videos for operative procedures were generated using Final Cut Pro 2022 (Apple, Cupertino, CA, USA). Figures for operative procedures were generated using Photoshop 2022 (Adobe, San Jose, CA, USA).	6
**RESULTS**		
10a	**Participants** – comprehensively describe:<br>• T To minimize selection bias related to surgical proficiency, only cases from surgical teams with the highest volume of thyroid cancer surgeries were included (OT: > 500 cases/year, RT: > 300 cases/year). The inclusion criteria were: 1) age of 18–65 years; 2) pathologically-confirmed DTC; 3) maximum lesion diameter ≤ 4 cm; and 4) undergoing total thyroidectomy and postoperative RAI therapy. The exclusion criteria were: 1) undergoing secondary surgery; 2) history of neck radiation therapy; 3) tumors found to invade critical structures, such as the esophagus, recurrent laryngeal nerve, major neck vessels, or trachea, on preoperative or intraoperative examination; 4) undergoing lateral neck dissection; and 5) preoperative routine examination revealing distant metastases. Details of the surgical procedures, patient selection flowchart, and RAI treatment are provided as online-only material supplementary (e Methods).	7
10b	**Participant comparison**<br>• To minimize selection bias related to surgical proficiency, only cases from surgical teams with the highest volume of thyroid cancer surgeries were included (OT: > 500 cases/year, RT: > 300 cases/year). The inclusion criteria were: 1) age of 18–65 years; 2) pathologically-confirmed DTC; 3) maximum lesion diameter ⩽ 4 cm; and 4) undergoing total thyroidectomy and postoperative RAI therapy. The exclusion criteria were: 1) undergoing secondary surgery; 2) history of neck radiation therapy; 3) tumors found to invade critical structures, such as the esophagus, recurrent laryngeal	8

	nerve, major neck vessels, or trachea, on preoperative or intraoperative examination; 4) undergoing lateral neck dissection; and 5) preoperative routine examination revealing distant metastases. Details of the surgical procedures, patient selection flowchart, and RAI treatment are provided as online-only material supplementary (e Methods).	
10c	**Intervention** – comprehensively describe: • none	
11a	**Outcomes** – comprehensively describe: • none	
11b	**Tolerance** – comprehensively describe: • none	
11c	**Complications** – comprehensively describe: Any episodes of tetany, transient and permanent hypoparathyroidism17, temporary and permanent hoarseness18, and postoperative bleeding19 were documented (Tables 5).	6
12	**Key results** – comprehensively describe: • In this study, we introduced dynamic risk assessment with 131I-WBS and assessed the efficacy of RAI therapy to compare surgical outcomes between the RT and OT groups. Our findings revealed no significant differences in the results of dynamic risk assessment before and after RAI therapy between the two groups, further confirming that the completeness of lesion resection in the RT group was similar to that of open surgery.	7-10
**DISCUSSION**		
13	**Discussion** – comprehensively describe: • Currently, open surgery remains the established standard treatment for thyroid cancer. However, it often results in noticeable neck appearance changes postoperatively, which can be particularly distressing, especially for young female patients, potentially leading to lifelong regret20. The da Vinci® robotic system, as an advanced endoscopic control device, has emerged as an alternative in thyroid surgery21. Nevertheless, there is an ongoing debate regarding whether the therapeutic outcomes of robotic surgery are comparable to those of open surgery22. In this study, we introduced dynamic risk assessment with 131I-WBS and assessed the efficacy of RAI therapy to compare surgical outcomes between the RT and OT groups. Our findings revealed no significant differences in the results of dynamic risk assessment before and after RAI therapy between the two groups, further confirming that the completeness of lesion resection in the RT group was similar to that of open surgery. • Analysis of demographic data revealed that patients in the RT group were, on average, younger compared to those in the OT group. This suggests that the scarless postoperative neck appearance associated with the RT approach provides better cosmetic outcomes and may be preferred by younger patients. Tumor diameters, staging, and locations were similar between the two groups, indicating similar tumor burdens and surgical complexities. However, the RT group had longer operative times for both TT + UCND and TT + BCND compared to the OT group. We attribute this primarily to the preparation time required for robotic surgery, including trocar placement, establishment of the initial operative space, and robotic arm connection, which typically takes about 30 minutes23. This resulted in longer overall operative times compared to open surgery, in line with previous research findings6. • Surgical efficacy evaluations often rely on single-point static assessments based on intraoperative or postoperative pathological and clinical data24. However, these assessments lack the ability to dynamically adjust based	10-14

14	on clinical data obtained during follow-up, limiting their effectiveness. The ATA dynamic risk stratification system, incorporating biochemical (Tg and TgAb) and imaging (131I-WBS, CT, and ultrasound) findings, provides a more comprehensive framework for assessment25. 131I-WBS, a routine imaging modality after RAI therapy, can be used to detect tiny residual or micro-metastatic thyroid cancer lesions often missed by other structural imaging modalities, including CT and ultrasound. Our study revealed a significant difference in dynamic risk assessment with and without 131I-WBS. Positive findings on 131I-WBS led many patients initially classified as ER to be reclassified as IDR or SIR. This highlights the importance of dynamic risk assessment with 131I-WBS for accurate evaluation of surgical efficacy.<br>• Furthermore, we assessed the surgical completeness of both methods by comparing multiple parameters. Although more lymph nodes were cleared in the OT group than in the RT group, there was no significant difference in the number of metastatic lymph nodes between the two groups. Additionally, high iodine uptake and increased radioactivity in the thyroid bed during technetium imaging and 131I-WBS provided evidence of remnant thyroid tissue. Through 3-hour iodine uptake, technetium imaging, and dynamic risk assessment with 131I-WBS at the time of RAI and after 6 months of RAI therapy, the RT group demonstrated outcomes similar to the OT group. This indicated that RT was as effective as OT in tumor eradication.<br>• Besides, the rates of parathyroid gland mis-resection and transplantation were lower in the RT group compared to the OT group. This may be attributed to the magnified 3D field of view and flexibility of Endo-Wrist® instruments in robotic surgery, which facilitated finer operations. Moreover, the RT group exhibited significantly lower rates of transient and permanent hypoparathyroidism compared to the OT group, which was consistent with previous reports from our team26. We analyzed that this was due to better protection of the parathyroid gland by preserving the true capsule behind the thyroid gland via SMCD. Previous studies have demonstrated that the SMCD technique7 in robotic surgery effectively preserves challenging-to-retain parathyroid gland types, including compact, intrathyroidal, and subcapsular parathyroid glands, along with their blood supply27, thereby reducing the incidence of parathyroid gland dysfunction28. Finally, during follow-up, ranging from 6 to 76 months, no difference was found in the recurrence rate between the two groups..	14
14	**Strengths and limitations** – comprehensively describe:<br>• The present study had several strengths. Firstly, we uniquely integrated 131I-WBS into a dynamic risk assessment framework, complemented by a postoperative evaluation of RAI therapy efficacy. This innovative method offered a nuanced understanding of the comparative efficacy and safety of RT and OT, providing crucial insights for treatment decision-making. Secondly, PSM allowed meticulous mitigation of biases arising from clinical and pathological variations between the two groups, enhancing the reliability and credibility of our conclusions and ensuring robust comparisons between the two approaches. Thirdly, the inclusion of a large and diverse observational cohort, reflecting real-world clinical practice, improved the generalizability of our findings. Through comprehensive long-term follow-up assessments, we not only recorded immediate postoperative outcomes but also elucidated the enduring impact of RT and OT on factors such as recurrence rates and parathyroid function. This holistic perspective offers valuable insights into the	14

	sustained efficacy and patient-centered outcomes associated with each surgical modality. Finally, our study addressed a critical gap in current research by comparing the safety and efficacy of RT and OT specifically for thyroid cancer, contributing significantly to informed clinical decision-making and ultimately improving patient outcomes and quality of life.<br>• However, the study also had several limitations. Firstly, the proficiency of RT in a single center may not represent the overall level for thyroid surgery. Moreover, the study used the UABA or BABA approach for RT, which may have different effects compared to other approaches. Secondly, patients requiring lateral neck dissections were not included, necessitating further research into the effectiveness of robot-assisted surgery for this subgroup. Thirdly, DTC generally has a favorable prognosis, and the limited follow-up time in this study may not reflect final treatment outcomes for all cases. Therefore, designing multicenter prospective studies with larger sample sizes, broader indications, and longer follow-up durations is essential to further validate the conclusions of this study.	
15	**Relevance and implications** – comprehensively describe:<br>• none	
**CONCLUSION**		
16	This study introduced a novel method by incorporating the dynamic assessment system of 131I-WBS with the efficacy of radioactive iodine therapy, allowing precise evaluation of the completeness of thyroid cancer surgery. Moreover, it indicated that RT through SMCD achieved efficacy comparable to OT while improving the protection of parathyroid function.	15
**DECLARATIONS**		
17a	**Conflicts of interest**<br>• none	15
17b	**Funding**<br>• none	15
17c	**Contributorship**<br>• The authors also thank all the patients included in this study.	15
